# Serum apolipoproteins in relation to intakes of fish in population of Arkhangelsk County

**DOI:** 10.1186/1743-7075-9-51

**Published:** 2012-06-08

**Authors:** Natalia Petrenya, Magritt Brustad, Marie Cooper, Liliya Dobrodeeva, Fatima Bichkaeva, Gulnara Lutfalieva, Jon Øyvind Odland

**Affiliations:** 1 Norwegian Institute of Food, Fisheries and Aquaculture Research, Muninbakken 9-13, Breivika, P.O. box 6122, N-9291, Tromsø, Norway; 2 Centre for Sami Health Research, Institute of Community Medicine, University of Tromsø, N-9037, Tromsø, Norway; 3 Institute of Environmental Physiology, Ural Branch, The Russian Academy of Sciences, 163061, Arkhangelsk, Russia; 4 Institute of Community Medicine, Faculty of Health Sciences, University of Tromsø, N-9037, Tromsø, Norway

**Keywords:** Serum lipids, Apolipoprotein A-I, Apolipoprotein B, Apolipoprotein B/ Apolipoprotein A-I ratio, Fish consumption, Nenets Autonomous Okrug, Arkhangelsk County

## Abstract

**Background:**

Diets rich in omega-3 fatty acids and low in saturated fat were found beneficially associated with blood lipids and cardio-vascular health. Lean reindeer meet and local cold water white-fish species high in omega-3 are among the main sources of nutrients in the rural area of the Nenets Autonomous Okrug (NAO) in Russia and are not normally consumed by the urban population from the same region. The aims of the study were firstly, to compare serum lipid profiles of residents of urban (Arkhangelsk city) and rural (NAO) regions of Arkhangelsk County, and secondly, to investigate the effects of fish consumption on the predictor of cardiovascular events apolipoprotein (Apo) B/ApoA-I ratio in these populations.

**Methods:**

A cross-sectional study conducted in Arkhangelsk County, Russia. Sample size of 249 adults: 132 subjects from Arkhangelsk city, aged 21–70 and 117 subject (87% Ethnic Nenets) from NAO, aged 18–69.

**Results:**

We observed more favorable lipid levels in NAO compared to Arkhangelsk participants. Age-adjusted geometric means of ApoB/ApoA-I ratio were 1.02 and 0.98 in men and women from Arkhangelsk; 0.84 and 0.91 in men and women from NAO respectively. Age and consumption of animal fat were positively associated with ApoB/ApoA-I ratio in women (pooled samples from Arkhangelsk and NAO). Body mass index and low levels of physical activity were positively associated with ApoB/ApoA-I ratio in men (pooled samples from Arkhangelsk and NAO). Reported oily fish consumption was not significantly correlated with ApoB/ApoA-I ratio.

**Conclusion:**

The population sample from rural NAO, consisting largely of the indigenous Arctic population Nenets with healthier dietary sources, had a relatively less atherogenic lipid profile compared to the urban Arkhangelsk group. Fish consumption had no effect on apolipoproteins profile.

## Background

Fish consumption is believed to have cardio-protective effects, attributed to long-chain n-3 polyunsaturated fatty acids (PUFAs), eicosapentanoic acid (EPA) and docosahexanoic acid (DHA) [1,2].

Fish-consuming populations have lower coronary heart disease (CHD) mortality rates than non-fish-consuming populations [3,4]. An observational study in Kohama island, Japan, reported that the population of the island had the lowest incidence of cardiovascular disease (CVD) in this country. They also had a higher serum level of eicosapolyenoic acids (46.8 mg/100 ml) than that in people on mainland Japan, owing to the higher intake of fresh fish (147.7 g/day). A positive correlation was found between serum EPA concentration and high-density lipoprotein cholesterol (HDL-C) level [5].

In the late 1970s, epidemiological studies by Bang and Dyerberg showed that Greenland Inuit had a lower risk of CVD compared to the Danish population due to low intake of saturated fatty acids and high intake n-3 PUFAs, derived from fish, seal and whale (polyunsaturated fatty acids / saturated fatty acids ratio was 0.84 in Inuit and 0.24 in Danes). Nearly double the intake of proteins in Inuit as compared to Danes, compensated for by a reduction in carbohydrates. Inuit were reported to have decreased blood concentrations of total cholesterol (TC), triglycerides (TG), low-density lipoprotein cholesterol (LDL-C) and very low-density lipoprotein cholesterol (VLDL-C) and men had also increased levels of HDL-C [6-11].

In the early 90s, a health survey conducted in the Inuit of Nunavik of Quebec of Canada, demonstrated that mean consumption of traditional food of marine origin (mattak (white whale skin), red char (arctic char), ringed seal meat, lake trout, lake white-fish and etc.) was 131 g/day, investigated by 24-h dietary recall in the total group and 163 g/day, investigated by food frequency questionnaire in women. Concentrations of n − 3 fatty acid in plasma phospholipids were positively associated with HDL-C concentrations and inversely associated with TG concentrations and the ratio of total to HDL cholesterol. In contrast, concentrations of TC, LDL-C and plasma glucose increased as n-3 fatty acid concentrations increased [12]. During the period 1992–1996, the age-standardized mortality rate for ischemic heart disease (IHD) was lower among Inuit (66.3/100,000) then among the non-indigenous Quebec population of Canada (140.2/100,000) [13]. In the early 90s, a similar study was conducted among the Inuit of Greenland. The intake of marine food was estimated to be 22% of the total energy and distributed as 100 g/day of seal, 64 g/day of fish, 44 g/day of whale and 17 g/day of sea-birds. A diet rich in marine oils was positively associated with serum HDL-C and blood glucose and inversely with VLDL-C and TG among this population [14].

Currently, apolipoprotein A-I (ApoA-I), apolipoprotein B (ApoB) and the ApoB/ApoA-1 ratio are established as more informative indicators of CVD than conventional serum lipids [15,16]. The ApoB/ApoA-I ratio is a measure of imbalance between atherogenic and anti-atherogenic lipoproteins. It is a strong predictor of myocardial infarction, stroke, heart failure and is also related to the risk of renal failure and aortic aneurysm. Based on results from the Apolipoprotein-related Mortality RISk (AMORIS) [17] and INTERHEART studies [18], ApoB/ApoA-I values above 0.9 in men and 0.8 in women indicate a high risk of myocardial infarction.

Russia is a country with high level of CVD death. While CVD-related deaths have decreased in Japan, North America and Western Europe during the last decades, the CVD deaths in Russia increased. According to the World Health Organization (WHO), in the European Union (27 countries) CHD mortality (age-standardized by world population) in men declined from 139/100,000 in 1985–1989 to 93/100,000 in 2000–2004 (33% decrease). In women, the fall was from 61/100,000 to 44/100,000 (27% decrease). In the Russian Federation, CHD mortality rates in 2000–2004 were exceedingly high, around 380/100,000 for men (23% increase) and 170/100,000 for women (2% increase) [19].

A prospective cohort study of 3704 adults performed in Arkhangelsk showed that ApoB/ApoA1-ratio was the strongest predictor of CVD and all-cause death in men and CVD death in women [20]. The data, collected at baseline of this study in 2000, demonstrated that metabolic syndrome prevalence was higher in women compared to men. The considerably higher prevalence was observed in women aged 50–59 (41%) and aged 60 or over (46%) [21]. Low levels of HDL-C, high systolic blood pressure and central obesity were the most frequent markers of metabolic syndrome in Arkhangelsk women [22]. In this study, hazardous alcohol consumption was an independent risk factor for CVD mortality in women [20].

Lean reindeer meet and local cold water white-fish species high in omega-3 fatty acids are among the main sources of nutrients in the rural area of the Nenets Autonomous Okrug (NAO) and not normally consumed by the urban Arkhangelsk population. We have previously shown that current median oily fish consumption was 19.5 g/day in both the rural NAO and the urban Arkhangelsk city population samples. Oily fish primarily consumed by the NAO subgroup was local self-caught white-fish species while market-bought fish, predominantly processed herring, was the most frequently consumed seafood item in the urban subgroup [23].

Arkhangelsk city is the administrative centre with a population of 348,740 (predominantly ethnic Russians) in 2008 [24]. The total number of people in NAO was 42,019 in the same year [24]. The indigenous village of Nelmin-Nos is located within the Arctic Circle, in the rural NAO. In 2008, the total population of the village aged 18 years or older was 580 people (282 women and 298 men). Nenets people constituted 93% of adult population. In 2008, the mean age of the urban population of the Arkhangelsk region was 34.7 years in men and 39.9 years in women, while the average age in NAO was 32.2 years in men and 35.7 in women [24].

The objectives of the current project were firstly, to compare serum lipid profiles of residents of urban (Arkhangelsk city) and rural (NAO) regions of Arkhangelsk county, and secondly, to investigate the effects of fish consumption on the predictor of cardiovascular events ApoB/ApoA-I ratio in these populations.

## Methods

### Study groups and design

It was not feasible to get an access to the population registers of the Arkhangelsk region, because of legal restrictions. The study subjects were therefore not selected randomly. Two communities: Arkhangelsk city and the settlement in the rural NAO area that consisted largely of indigenous Arctic population Nenets were chosen for data collection.

For recruitment, we chose some institutions in Arkhangelsk city where female workers were predominant (scientific institute, library, retail and clothing industry) and both verbal and written invitations to the health screening were used. In addition, some enrollment was achieved through invitation letters distributed to mailboxes of block housing in the vicinity of our research centre in Arkhangelsk.

In Nelmin-Nos, the recruitment to the study was primarily through advertisement of the health screening placed in various public areas (medical centre, shops, school, school canteen, kindergarten, museum, public bath house) and also through verbal invitation by local health professionals. Similarly to the strategy used in Arkhanglsk city, the places for advertisement were chosen on the basis of a predominance of female workers.

Participants from Arkhangelsk were invited to visit the Institute of Environmental Physiology and participants from Nelmin-Nos were invited to visit the medical center in their settlement. The screening consisted of physical examination, blood sampling and questionnaire survey. The study in Arkhangelsk city was conducted between April 2008 and April 2009. Field work in the village of Nelmin-Nos was performed in February 2009.

The entire sample consisted of 300 subjects between 18 and 77 years of age (54 males and 246 females). In total, 28 men (16.9%) and 138 women (83.1%) from Arkhangelsk city and 26 men (19.4%) and 108 women (80.6%) from the village of Nelmin-Nos were enrolled.

The statistical analysis showed the differences in socio-economical status when the two residences were compared. The samples from Arkangelsk and Nelmin-Nos were well matched according to age- and gender- distribution [23].

Pregnant women, subjects taking cholesterol-lowering medications and with self-reported diabetes, coronary heart disease and stroke were excluded from the present analysis. In addition, four subjects from Arkhangelsk city and five subjects from the village of Nelmin-Nos had missing questionnaire data. The total number of participants after exclusion was 249. The rationale for excluding subjects (34 individuals (20.5%) from the Arkhangelsk sample and 17 individuals (12.7%) from the Nelmin-Nos sample was to control for potential confounders.

Medical history and lifestyle information were collected via research staff-administrated (59.1% for Arkhangelsk and 25.2% for Nelmin-Nos) or self-administrated (40.9% for Arkhangelsk and 74.8% for Nelmin-Nos) questionnaires. The same questionnaire was applied for both methods.

### Ethics approval

The study was approved by the Ethical Committee at the Northern State Medical University, Arkhangelsk. Written informed consent was obtained from each participant.

### Ethnicity, lifestyle and dietary factors

Four questions on ethnicity were developed based on a Russian validated version of the Survey of Living Conditions in the Arctic: Inuit, Saami, and the Indigenous Peoples of Chukotka study (SLiCA study) [25]. Ethnicity was defined as Nenets if either the mother or father of the participant considered themselves to belong to Nenets and in addition participant was Nenets by documents and considered themselves Nenets. The majority of NAO participants had ethnic Nenets for parents (68% of men and 82.6% of women from NAO). Some younger people had mixed origin, for example, the father was of Russian origin or mixed aboriginal and non aboriginal origin and mother was of Nenets origin (16% of men and 5.4% of women from NAO). Ethnicity was defined as Russians/Other (for example, Ukrainian, Byelorussian, Komi) if mother and father of the participant considered themselves to belong to Russians/Other and in addition the participant was Russian/Other by document and considered themselves Russians/Other (100% of men and women from Arkhangeslk and 16% of men and 12% of women from NAO). The majority of Arkhangesk participants had ethnic Russian parents.

Physical activity was categorized as sedentary, moderate or high. A sedentary job and leisure lifestyle were defined as sedentary physical activity. Walking and other physical activity, such as light gardening of at least 4 h per week during leisure or work were defined as moderate physical activity. High physical activity included sports, such as jogging, skiing, gymnastics, swimming or intensive gardening or its equivalent for at least 4 h a week.

Daily, or occasional smokers, or those who quit less than two years ago were categorized as current smokers. Ex- or never smokers were recorded as non-smokers.

Fish consumption in grams/day was estimated using a food frequency questionnaire. The method is described in detail elsewhere [23].

Questions related to portion size were asked separately for boiled and fried fish meals: one slice (150 gram), one and a half slices (225 gram), two slices (300 gram), three + slices (450+ gram).

Information about portion size for the first fish item (boiled cod, haddock and saithe) was derived from the question for boiled fish meal and information about portion size for the second fish item (fried cod, haddock and saithe) was derived from the question for fried fish meal.

For three items: 1) wolfish, flounder, redfish, 2) whitefish and 3) other freshwater fish species portion size was calculated as an average of boiled and fried portions(boiled + fried)/2.

We assumed the standard portion of 150 gram for four fatty fish items: 1) salmon (trout), 2) mackerel, 3) herring and 4) pink salmon, because these fatty fish species are consumed in a wide variety of ways and not predominantly as boiled or fried fish meal.

Fish was categorized as oily (= fatty fish) when it’s fat content exceeded four percent. The fatty fish species commonly consumed were salmon, trout, mackerel, herring, pink salmon and local to NAO white-fish species *(Coregonus lavaretus, Coregonus peled, Coregonus nasus, Stenodus leucichthys nelma).* In addition, participants from Arkhangelsk were asked how often they ate fatty fish as a main dish: never/seldom, once a month, 2–3 times a month, once a week, two or more times a week. Saturated fat consumption in grams/day was estimated from the intake of meat dishes: including soups based on meat/chicken broth. We calculated the average amount of fat (gram per portion) for all listed food items according to the values provided in the National Russian food composition tables [26]. Standard portions of 75 g for meat, chicken and meat rissoles/cakes, 50 g for canned meat, 100 g for sausages and 250 g for soup were used.

With respect to alcohol consumption participants were asked the following questions: how much did you drink during the last 7 days and how often and how much have you drunk on average in the last twelve months? Categories were: never/seldom, 1 per month, 2–3 per month, 1 per week, 2–4 per week, 5–6 per week. The questions also addressed the type of alcoholic beverage consumed. The daily and weekly number of alcohol units (AU) consumed was calculated. One AU corresponded to 13.8 g of pure alcohol.

We assessed alcohol problems by a formerly used Russian version of the CAGE-questionnaire [27]. Participants replied yes or no to four specific questions. Summary scores were calculated across responses and two or more positive answers were taken as the cut-off point for problem drinking.

### Anthropometric measurements

Body weight in kg (± 50 g) was measured with participants wearing light clothing using an electronic scale (A&D UC-322, Japan). Height was measured to the nearest 0.1 centimeter using a standard stadiometer. Body mass index (BMI) in kg/m^2^ was calculated.

### Laboratory measurements

Fasting venous blood samples were collected using vacutainers and centrifuged within 30 minutes. Serum was aliquotted and stored frozen at −20°C. The samples from Nelmin-Nos were drawn by the same staff, stored frozen and transported to Arkhangelsk city. Measurements were performed at the laboratory of Biochemistry at the Institute of Environmental Physiology with an automated clinical biochemical analyzer « MARS » (Infopia Co, Ltd, Anyang, Korea) or « Cary 50 » spectrophotometer (Australia). Reagents from "Chronolab AG" (Switzerland) were used. Total Cholesterol, HDL-C and TG were measured by enzymatic-colorimetric tests. Low-density lipoprotein cholesterol was measured by a turbidimetric method as previously described by Burstein and Samaille [28,29]. Very low-density lipoprotein cholesterol was calculated with the Friedewald Equation where VLDL-C = triglyceride/5. Apolipoprotein A-I and apoB were assayed by an imminoturbodimetric method with polyclonal goat serum anti-human apolipoprotein antibodies ("Chronolab AG"). The assay and calibrator concentration have been standardized against the WHO/IFCC SPI/0I standard for ApoA-I and the WHO/IFCC SP3/07 for ApoB (CDC, USA). Both external and internal quality controls were established. Measurements met the standards of international Quality Assurance/Quality Control network. The analytic covariance for all parameters was ≤3%.

### Statistical analysis

Because Shapiro-Wilk test showed skewed distribution for all the serum lipids, these variables were log10-transformed prior to statistical analyses. The fish intake variable was log10(x + 1)-transformed to correct for skewed distribution.

Analysis of covariance (ANCOVA) was performed to compare mean values of biochemical markers adjusted for age in two populations with respect to gender. Means and confidence intervals for all parameters are presented in original units.

Multiple linear regression analyses were used to assess associations between ApoB/ApoA-I ratio and age (continuous variable), residence (dichotomous variable), BMI (continuous variable), physical activity (two dummy variables, the reference category (0) was high level of physical activity), smoking (dichotomous variable), fat consumption from meat (continuous variable), alcohol consumption (continuous variable) or alcohol dependence when appropriate (polychotomous variable: 0 indicated CAGE score 0–1, 1–3 indicated CAGE score 2–4) and fish intake (continuous variable) separately in men and women (pooled samples from Arkhangelsk and NAO).

Since oily fish consumption and ApoB/ApoA-I did not show a linear relationship in women (pooled samples from Arkhangelsk and NAO and sample from Arkhangelsk), a second order quadratic term was used in the model. This model was found more appropriate due to observed non-linear reverse U-shape association between ApoB/ApoA-I ratio and tertiles of oily fish consumption.

The best predicting models with forward stepwise linear regression analyses were performed subsequently separately for men and women.

We presented β and p-values for independent variables of interest and R^2^ and p-value for model.

A p-value of ≤ 0.05 (two-tailed tests) was defined as significant. Statistical analysis was performed using SPSS for Windows statistical package (version 19.0; SPSS Inc. Chicago, IL, USA).

## Results

Age distribution in males and females before and after exclusion by residence in comparison with official statistical data is shown in Table 1 and Table 2.

**Table 1:** Age distribution in males before and after exclusion by residence in comparison with official data

**Age groups (%)**	**Arkhangelsk region urban population 2008**	**Arkhangelsk city entire sample April 2008-April 2009**	**Arkhangelsk city present study sample**	**Nelmin-Nos population 2009**	**Nelmin-Nos entire sample February 2009**	**Nelmin-Nos present study sample**
	**n 323701#**	**n 28**	**n 21, excluded n 7**	**n 268**	n 23*	n 22*, excluded n 1
20-29	26.8	21.4	14.3	23.9	26.1	27.3
30-39	21.3	28.6	38.1	25.4	26.1	27.3
40-49	20.0	25.0	28.6	25.7	21.7	22.7
50-59	18.3	14.3	9.5	17.5	21.7	18.2
60+	13.6	10.7	9.5	7.5	4.4	4.5
Total	100.0	100.0	100.0	100.0	100.0	100.0

*Three males from Nelmin-Nos (age group 18–19 years old) were not included in the present table.

#36.2% of males are Arkhangelsk residents.

**Table 2:** Age distribution in females before and after exclusion by residence in comparison with official data

**Age groups (%)**	**Arkhangelsk region urban population 2008**	**Arkhangelsk city entire sample April 2008-April 2009**	**Arkhangelsk city present study sample**	**Nelmin-Nos population 2009**	**Nelmin-Nos entire sample February 2009**	**Nelmin-Nos present study sample**
	**n 405329#**	**n 138**	**n 111, excluded n 27**	**n 285**	n 107*	n 91*, excluded n 16
20-29	21.5	9.4	9.0	20.4	14.0	14.3
30-39	17.3	18.1	22.5	24.9	24.3	25.2
40-49	18.0	17.4	17.1	20.7	22.4	25.3
50-59	19.9	42.8	43.2	21.4	26.2	27.5
60+	23.3	12.3	8.2	12.6	13.1	7.7
Total	100.0	100.0	100.0	100.0	100.0	100.0

*One female from Nelmin-Nos (age group 18–19 years old) was not included in the present table.

#38.3% of females are Arkhangelsk residents.

Antropometric and lifestyle characteristics of the study participants are shown in Table 3. The urban Arkhangelsk population was slightly older than Nelmin-Nos participants when both genders were pooled (p = 0.022). When samples were stratified by gender the age difference remained among women only (p = 0.044).

**Table 3:** Anthropometric, lifestyle and dietary characteristics of the study participants (n 249)*

**Characteristics**	**Males**		**p-value**	**Females**		**p-value**
	**Arkhangelsk (n 21)**	**Nelmin-Nos (n 25)**		**Arkhangelsk (n 111)**	**Nelmin-Nos (n 92)**	
Age (years)^†^	38.0	36.0	0.310^a^	50.0	46.0	0.044^a^
Ethnic groups (%)^§,^						
Russians/Other	100.0	16.0	<0.0001^b^	100.0	12.0	<0.0001^b^
Nenets	0.0	84.0		0.0	88.0	
Education (%)^§^						
Incomplete secondary	9.6	41.7	<0.0001^b^	4.6	14.6	<0.0001^b^
Complete secondary ^||^	33.3	54.2		55.0	76.4	
Complete high, post grad	57.1	4.1		40.4	9.0	
Full-time employee (%)^§^						
Yes	81.0	54.2	0.057 ^b^	78.6	65.6	0.042 ^b^
No	19.0	45.8		21.4	34.4	
BMI (kg/m^2^)^‡^	27.4	24.7	0.025^d^	26.6	28.1	0.060^d^
Smoking status (%)^§^						
Current	42.9	50.0	0.632^b^	15.7	16.9	0.830^b^
Never, ex smokers	57.1	50.0		84.3	83.1	
Physical activity (%)^§^						
Low	23.8	11.1	0.130^c^	33.7	39.4	0.707^b^
Moderate	38.1	16.7		46.2	43.7	
High	38.1	72.2		20.1	16.9	
CAGE-test score (%)^§^						
0-1	89.4	68.7	0.460^c^	97.9	74.5	<0.0001^c^
2	5.3	12.5		2.1	12.7	
3	5.3	12.5		0.0	4.3	
4	0.0	6.3		0.0	8.5	
Alcohol U/last 7 days (AU)^†^	5.0	2.4	0.011^a^	1.0	0.0	<0.0001^a^
Alcohol U/week last year (AU)^†^	1.0	0.6	0.132^a^	0.6	0.2	0.037^a^
Fat from meat (g/d)^†^	15.6	16.9	0.391^a^	12.8	10.4	0.046^a^
Total fish (g/d)^†^	81.6	44.9	0.145^a^	41.1	24.9	0.002^a^
Oily fish (g/d)^†^, **	39.9	42.3	0.842^a^	17.4	20.0	0.149^a^
Lean fish (g/d)^†^	49.8	0.0	<0.0001^a^	23.6	0.0	<0.0001^a^
Local oily fish (NAO) (g/d)^†^	0.0	37.4	<0.0001^a^	0.0	14.0	<0.0001^a^

^a^ Mann–Whitney U test. ^b^ Pearson Chi-Square test. ^c^ Fishers Exact test. ^d^ ANCOVA test.

^*^ Subgroups are not total 249 because of missing values, except age and ethnic groups.

^†^ Age, alcohol U/last 7 days, alcohol U/week during last year, fat from meat, total fish, oily fish, lean fish and local oily fish variables are presented as median.

^‡^ BMI is presented as geometric mean adjusted for age.

p-value.^§^ Ethnic groups, education, full-time employee, smoking status, physical activity and CAGE-test score variables are presented as percentage from total number within residence.

^||^ Includes secondary, professional secondary and incomplete high education.

^**^ Includes local oily fish (NAO).

Men from Nelmin-Nos had lower BMI, compared to men from Arkhangelsk. Among women, BMI was higher in Nelmin-Nos, but not significantly. We found no differences in smoking habits in either men or women from the two populations. Nearly half of men were defined as current smokers. Among women, 15.7% from Arkhangelsk city and 16.9% of women from NAO reported smoking. Men from the village of Nelmin-Nos were more physically active; however, the difference in physical activity between men was not statistically significant. In both populations, one-third of women had low levels of physical activity.

Twenty seven percent of participants from Nelmin-Nos and 3.5% from Arkhangelsk had alcohol problems according to the screening test (CAGE test) (p < 0.0001). When samples were stratified by gender, we observed that the difference was significant among women only (p < 0.0001). However, women from Nelmin-Nos reported significantly lower alcohol intake either when estimated as consumption during the last seven days (p < 0.0001) or as AU consumed per week during last year (p = 0.037). Men from Nelmin-Nos reported a significantly lower alcohol consumption during the last 7 days (p = 0.011) compared to participants from Arkhangelsk. There was a substantial amount of missing data on alcohol consumption and CAGE test comprising 14.4% (AU last seven days), 17.4% (AU per week during last year) and 12.1% (CAGE score) for Arkhangelsk vs. 33.3%, 34.9% and 40.9% respectively for Nelmin-Nos.

Women from NAO reported lower animal fat (p = 0.046) and total fish consumption (p = 0.002) calculated in g/day. When participants from Arkhangelsk were asked how often they ate fatty fish as a main dish, 51% replied never/seldom, 16.7% - once a month, 14.8% - 2–3 times a month, 8.7% - once a week and 8.8% - two or more times a week. Fatty fish consumption in g/day calculated from the type of fish question correlated positively (r = 0.329, p < 0.0001, n = 149) with fatty fish consumption as the main dish.

Age-adjusted geometric means of biochemical markers were compared across residences and in relation to gender. The data are presented in Table 4. The proportion of men having ApoB/ApoA-I ratio of 0.9 and higher was 52.4% for Arkhangelsk and 47.6% for Nelmin-Nos (p = 0.172). The proportion of women having ApoB/ApoA-I ratio of 0.8 and higher was 58.3% for Arkhangelsk and 41.7% for Nelmin-Nos (p = 0.086).

**Table 4:** Serum lipids* in population samples from Arkhangelsk and Nelmin-Nos (n 249)

**Parameters**	**Males**			**Females**		
	**Arkhangelsk (n 17–19)**	**Nelmin-Nos (n 25)**	**p-value**^**†**^	**Arkhangelsk (n 106–108)**	**Nelmin-Nos (n 90–91)**	**p-value**^**†**^
TC	5.3 (4.6-6.1)	4.5 (4.0-5.0)	0.068	5.8 (5.6-6.1)	5.0 (4.8-5.2)	<0.0001
HDL-C	0.9 (0.8-1.0)	1.2 (1.0-1.3)	0.005	1.2 (1.1-1.3)	1.2 (1.1-1.3)	0.857
LDL-C	5.0 (4.2-6.0)	3.8 (3.2-4.4)	0.019	4.4 (4.1-4.7)	4.5 (4.2-4.8)	0.691
VLDL-C	0.25 (0.19-0.32)	0.16 (0.13-0.2)	0.010	0.22 (0.2-0.24)	0.2 (0.18-0.22)	0.128
TG	1.2 (1.0-1.6)	0.8 (0.7-1.0)	0.010	1.1 (1.0-1.2)	1.0 (0.9-1.1)	0.183
ApoA-I	95.9 (88.5-103.8)	103.0 (96.2-110.4)	0.178	107.7 (102.8-113.0)	103.5 (98.2-109.0)	0.255
ApoB	98.0 (86.7-110.7)	86.9 (78.2-96.6)	0.148	105.2 (98.9-112.2)	94.4 (88.1-100.9)	0.022
ApoB/ApoA-I ratio	1.02 (0.88-1.18)	0.84 (0.74-0.96)	0.057	0.98 (0.90-1.06)	0.91 (0.83-0.99)	0.228

Abbreviations: TC, total cholesterol (mmol/L); HDL-C, high-density lipoprotein cholesterol (mmol/L); LDL-C, low-density lipoprotein cholesterol (g/L); VLDL-C, very low-density lipoprotein cholesterol (mmol/L); TG, triglyceride (mmol/L); ApoA-I, apolipoprotein A-I (mg/dL); ApoB, apolipoprotein B (mg/dL).

^*^The data are presented as adjusted geometric mean (95% confidence interval).

^†^P-value was calculated by ANCOVA (analysis of covariance) adjusted for age.

Age (β = 0.372, p < 0.0001) and fat from meat (β = 0.233, p = 0.001) predicted significantly ApoB/ApoA-I ratio in pooled samples of women from Arkhangelsk and NAO (R^2^adj. = 0.147, p < 0.0001, n = 184). Oily fish consumption was not statistically significant associated with ApoB/ApoA-I ratio in females.

Body mass index (β = 0.531, p < 0.0001) and the low level of physical activity (β = 0.385, p = 0.008) predicted significantly ApoB/ApoA-I ratio in pooled samples of men from Arkhangelsk and NAO (R^2^adj. = 0.333, p < 0.0001, n = 36). We have not found any associations between oily fish consumption and ApoB/ApoA-I ratio in males.

## Discussion

This study provides the preliminary results on distribution of serum lipids and apolipoproteins in the unique remote rural population of NAO, where the majority of inhabitants are Nenets indigenous people.

The main finding of this study was that participants from NAO had a relatively favorable lipid profile compared to participants from Arkhangelsk. Women from NAO had lower levels of TC and ApoB levels. The ApoB/ApoA-I ratio was 0.84 in men from NAO vs. 1.02 in men from Arkhangelsk. The difference however did not reach significance after adjustment for age. The conventional serum lipids were more favorable in men from NAO: HDL-C was higher, while VLDL-C, LDL-C and TG were significantly lower.

The CVD risk factors in Arctic Indigenous people have been intensively studied in recent decades. In the late 1970s, epidemiological studies demonstrated that Greenland Inuit had a lower risk of CVD compared to the Western population due to a diet rich in n-3 PUFAs [6-9]. Since the 1970s the Greenlandic diet has changed to include an increasing amount and variety of imported foods and a decreasing amount and variety of local foods [30]. More recent studies have shown that the prevalence of markers for CHD among Greenland Inuit was not different from that in Western populations. The highest prevalence was observed in the least westernized areas in Greenland [31]. The population survey conducted in 1998–2002 among Greenland Inuit concluded that the effect of westernization within communities in Greenland (to some extent due to dietary changes) on HDL-C and LDL-C was unfavorable for cardio-vascular health. Compared with the general population of Denmark, TC was higher among Inuit women, while HDL-C was higher among Inuit men. Triglycerides were lower among Inuits of both sexes [32].

The diet of Alaska Natives, including Inuit, American Indians and Aleuts traditionally consisted of foods rich in n-3 PUFAs. The mean daily intake of fish and shellfish for Alaska Natives was 109 g in 1987–1988 when the national intake was 17 g [33]. An autopsy study by McLaughlin *et al.* (1989–1993) in a sample of Alaska Native and non-Native subjects indicated that Alaska Natives had less advanced atherosclerosis in coronary arteries, along with higher proportions of n-3 PUFAs and lower proportions of n-6 PUFA in adipose tissue, than did non-Natives. The positive association was observed between HDL-C and long-chain n-3 adipose tissue triglyceride fatty acids in Alaska Natives [34]. A recent study of the Alaskan indigenous population showed that the prevalence of low HDL-C and high LDL-C was higher than among the US population with an overall prevalence of high cholesterol among Alaskan participants of 40%. It was concluded that most Alaskan Natives are now more sedentary and have transitioned to a mixed traditional and Western diet, thus the high prevalence of dyslipidemia is likely to continue [35].

Only few studies have investigated ApoB/ApoA-I ratio distribution among indigenous populations. A study from 1982 observed that native Greenland Inuits (healthy volunteers) had higher plasma ApoA-I and lower ApoB levels than Danes [36]. The ApoB level and ApoB/ApoA-I ratio were found to be higher in migrant South Asian Indians than in native Caucasians in Canada [37]. Apolipoprotein and lipid profiles in the First Nation population of Canada suggested high cardiovascular risk. Young Canadian women had ApoB/ApoA-I of 0.69, middle aged - 0.75-0.81 and old - 0.83. Young Canadian men had ApoB/ApoA-I of 0.71, middle-aged - 0.85-0.97 and old - 0.88 [38]. Judging by the level of ApoB/ApoA-I ratio, the ethnic Norwegian population from the population based study of health and living conditions in areas with both Sami and Norwegian populations (SAMINOR study), had a lower risk for CVD compared to the Russian population, whilst the Sami population of Norway, both men and women, had a lower risk for CVD compared to the Nenets population [39].

In our study oily fish consumption was not associated with ApoB/ApoA-I ratio in males and disappeared after controlling for confounders in females. Total and lean fish consumptions also did not correlate with ApoB/ApoA-I ratio in either of the populations studied (data not shown). Oily fish consumption was not different across residences. However, the types of oily fish used varied across communities. Residents of the indigenous village consumed predominantly locally caught fatty fish, termed “white-fish”. Residents from Arkhangelsk city consumed fatty fishes bought from food stores with herring reported as the most frequently consumed fatty fish (data not shown).

In the general population 250 mg of EPA and DHA per day appears sufficient for primary prevention of risk of coronary death. This corresponds to one 6-oz (170 g) serving (or 2 medium size servings) per week of wild salmon (approx. 1774 EPA + DHA mg per 6-oz serving). For individuals with IHD intake of 500–1000 mg per day appears reasonable for secondary prevention of risk of coronary death [2]. The content of n-3 PUFA in seafood varies considerably in relation to location and season of capture (mackerel 1.8-5.3 g / n-3 PUFA / 100 g, herring 1.2-3.1 g / n-3 PUFA / 100 g, salmon 1.0-2.0 g / n-3 PUFA / 100 g, trout 0.5-1.6 g / n-3 PUFA / 100 g) [40]. Lake white-fish (mixed species) were reported between 1 and 1.5 g / EPA + DHA / 100 g raw fish tissue [41]. In our study oily fish consumption was 121.8 g / week and 140 g / week among Arkhangelsk and Nelmin-Nos women respectively; 279.3 g / week and 296.1 g / week among Arkhangelsk and Nelmin-Nos men respectively.

It is likely that a high proportion of the oily fish eaten by subjects from Arkhangelsk sample was consumed in the form of salted, pickled, smoked, dried or canned preparations. Only 17.5% participants reported eating fatty fish as the main dish at least once a week. An unexpected finding was lower than anticipated total fish intake in the population sample from NAO. Women from Arkhangelsk had higher total fish consumption when compared to women from NAO. Only few people among the participants reported taking fish oil supplements, but not on a regular basis (data not shown). The estimation of the percentage of people with n-3 PUFA deficiency would be important in the populations studied. Based on long-term research and observation, fish consumption is considered to be a healthy choice and should be adequately presented in diet. Fish is a good source of high-quality proteins, vitamins D, A, B, minerals (calcium, phosphorus and iron) and trace elements (selenium, iodine and zinc).

The main effect of n-3 fatty acids on blood lipids profile was shown to lower triacylglycerols [42,43].

A systematic review of intervention studies [44] demonstrated that effects of omega-3 fatty acids on ApoA-I levels were generally heterogeneous, but small. Most of 27 studies found a small net change in ApoA-I with omega-3 fatty acid consumption. Three-quarters of studies found net changes between −5% and +5% (−7 to +10 mg/dL). No study found a large net increase in ApoA-I level. A small number of studies found larger net decreases of up to 18% reductions (−33 mg/dL). Little consistency in the effect of omega-3 fatty acids on ApoB levels was reported. About half the 25 studies found a small net increase and half a small net decrease in ApoB levels. One study found a significant decrease and one found a significant increase in ApoB levels.

Age was positively associated with ApoB/ApoA-I ratio in our study. In the large Swedish cohort the increase of ApoB/ApoA-I ratio continued from age 20 and was the highest in males aged 50–59 years old and in females aged 60–69 years old [45]. In our study we adjusted for age as the potential confounder.

It is possible that older people who had an adverse lipid profile in middle-age died prematurely and were underrepresented in NAO sample. The rural NAO was characterized in 2007 as an area with very low life expectancy (48.2 y for men and 65.9 y for women). For the year 2007, the life expectancy in the Arkhangelsk was 61.4 for men and 74.2 for women [24].

We found that a low level of physical activity was associated with a higher ApoB/ApoA-I ratio in men from both residences. Furthermore, BMI correlated positively with ApoB/ApoA-I ratio in men. The prevalence of obesity and physical inactivity should be of concern. American Indian and Alaskan Native people with higher levels of activity had significantly better clinical characteristics (HDL-C, TG, BMI and waist circumference) [46].

Fat consumption from meat dishes was positively associated with ApoB/ApoA-I ratio. Type of fat is believed to play a more important role in determining the risk of CHD than the total amount of fat in the diet [47]. Consumption of processed meat has been found to relate to a higher incidence of CHD and diabetes mellitus [48].

It is well known that the consumption of moderate doses of alcohol leads to the increase of HDL-C and ApoA-I [49]. The study of CVD risk factors and distribution of apolipoproteins in the urban population of Arkhangelsk conducted in 2000 showed a generally high level of serum ApoA-I. Frequency and quantity of alcohol consumed were important predictors of ApoA-I level in both sexes [50]. In our study, we found approximately the same level of ApoB, but ApoA-I was somewhat lower in both urban Arkhangelsk and rural NAO populations compared to data reported by Averina M. et al. [50]. We did not find any associations between ApoB/ApoA-I ratio and the amount of alcohol consumed. Excessive alcohol consumption appears to be an important risk factor for CVD death in the Arkhangelsk population. A recent follow-up study demonstrated that women reporting consumption of at least 80 g of alcohol monthly and consumption of 5 or more AU during one drinking episode had a greater risk of cardiovascular death than abstainers. This study also assessed a 10-year CVD and all-cause mortality risk in relation to ApoB/ApoA-ratio in the population of Arkhangelsk, and found that the ratio was among the strongest predictors of CVD mortality compared to other studied factors in both genders [20]. Lower alcohol consumption and higher GAGE scores in Nelmin-Nos participants could also be explained by genetic differences in alcohol tolerance between the ethnic groups of Mongoloid and Caucasian origin [51-55].

### Strength of the study

The strength of the present study was that all the samples were drawn in the morning after overnight fasting and were analyzed in the same laboratory using standardized laboratory techniques and kits. Moreover, the study provides clues on distribution of ApoA-I and ApoB in the remote rural population of NAO, where majority of inhabitants are the indigenous Nenets.

### Methodological limitations of the study

The sample size was relatively small and could result in attenuated statistical power, wider confidence intervals or risks of errors in analysis. In addition, we excluded 17% of the sample (pregnant women, subjects taking cholesterol-lowering medications, subjects with self-reported diabetes, coronary heart disease and stroke, subjects with missing questionnaire data). This exclusion could artificially skew the results and reduced the sample size of our study. However, using the method of exclusion we were able to avoid impact of potential confounders on results in our study. In addition, the proportions of excluded people from both samples were approximately equal.

Another limitation is the difficulty in obtaining a representative sample due to the lack of a population registry available for research. This could reduce an external validity and generalizability of our findings.

Young subjects were underrepresented in this sample. However, the deviations from official average age were similar for both residences. Median age in Arkhangelsk was 3.3 years over in men and in 10.1 years over in women than officially reported for the urban population of the Arkhangelsk region by the year 2008. Median age in Nelmin − Nos was 3.8 years over in men and in 10.3 years over in women than officially reported for the NAO population by the year 2008.

There were fewer face-to-face interviews in the Nelmin − Nos group. This could result in recall bias, especially on sensitive information regarding alcohol intake, tobacco use and physical activity level. A higher proportion of missing data on alcohol consumption was observed in NAO, compared to Arkhangelsk.

In our study vodka was the most frequently consumed alcohol beverage among males and table wine was the most frequently consumed alcohol beverage among females (data not shown). The actual consumption of alcohol and tobacco could not be optimally assessed using only the questionnaire. One more limitation of our study is that we did not measure laboratory markers of excessive alcohol consumption such as, gamma-glutamyl transferase that would supplement information obtained through the CAGE test. Our results on alcohol consumption and tobacco use should be interpreted with caution as they might be biased due to under-reporting. Many participants avoided answering alcohol and tobacco related questions.

The questionnaire on fat consumption from meat dishes was not validated. The questions were composed similarly to the validated Norwegian Women and Cancer Study (NOWAC study) [56-58], but modified to match the conditions and traditional food items of the Russian North West.

We have not calculated the total energy from the diet. It is likely that participants who reported eating high amounts of fatty fish consumed more food and more calories per day in general, which resulted in a higher ApoB/ApoA-I ratio. However, we calculated the amount of fat consumed from meat dishes and adjusted our models for this variable.

The method used to assess fish consumption and diet is discussed elsewhere [23]. As we were unable to adjust for all possible confounders it is likely that dietary and lifestyle factors might have influenced our results. For example, unmeasured binge drinking, differences in socio-economic status, psychosocial distress or low fruit and vegetable consumption would affect the study findings.

## Conclusions

Our results suggest that the rural NAO population of the Arkhangelsk region have a relatively favorable lipid profile when compared to the urban Arkhangelsk population. Age, BMI, physical activity and the amount of fat consumed from meat dishes were predictors of the ApoB/ApoA-I ratio. Fish consumption had no effect on apolipoproteins profile. More detailed analysis of dietary data is needed.

## Abbreviations

PUFAs: Polyunsaturated fatty acids; EPA: Eicosapentanoic acid; DHA: Docosahexanoic acid; CHD: Coronary heart disease; CVD: Cardiovascular disease; HDL-C: High-density lipoprotein cholesterol; TC: Total cholesterol; TG: Triglyceride; LDL-C: Low-density lipoprotein cholesterol; VLDL-C: Very low-density lipoprotein cholesterol; IHD: Ischemic heart disease; ApoA-I: Apolipoprotein A-I; ApoB: Apolipoprotein B; WHO: World Health Organization; NAO: Nenets Autonomous Area; AU: Alcohol unit; BMI: Body mass index; ANCOVA: Analysis of covariance.

## Competing interests

The authors declare that they have no competing interests.

## Authors’ contributions

NP designed the study, collected the data, performed statistical analysis and wrote the manuscript. MB participated in the design of the study, advised on statistical analysis, contributed to the draft and critical revision of the paper. MC planned the study and contributed to the draft and critical revision of the paper. LD coordinated the project, participated in the design of the study and collection of data. FB contributed to the data collection, laboratory measurements and provided advice on methodology. GL participated in collection of data and physical examination of the study participants. JØO supervised the project, participated in the design of the study, contributed to the draft and critical revision of the manuscript. All authors were involved in the discussion of the data, read and approved the final text. JØO has given final approval of the version to be published.
